# Association of in utero HIV exposure with child brain structure and language development: a South African birth cohort study

**DOI:** 10.1186/s12916-024-03282-6

**Published:** 2024-03-22

**Authors:** Catherine J. Wedderburn, Shunmay Yeung, Sivenesi Subramoney, Jean-Paul Fouche, Shantanu H. Joshi, Katherine L. Narr, Andrea M. Rehman, Annerine Roos, Diana M. Gibb, Heather J. Zar, Dan J. Stein, Kirsten A. Donald

**Affiliations:** 1 Department of Paediatrics and Child Health, Red Cross War Memorial Children’s Hospital, University of Cape Town, Cape Town, South Africa; 2 Department of Clinical Research, London School of Hygiene & Tropical Medicine, London, UK; 3 The Neuroscience Institute, University of Cape Town, Cape Town, South Africa; 4 Department of Psychiatry & Mental Health, University of Cape Town, Cape Town, South Africa; 5 Departments of Neurology, Psychiatry and Biobehavioral Sciences, University of California Los Angeles, Los Angeles, CA USA; 6 Department of Bioengineering, University of California Los Angeles, Los Angeles, CA USA; 7 MRC International Statistics & Epidemiology Group, London School of Hygiene & Tropical Medicine, London, UK; 8 SA MRC Unit On Risk and Resilience in Mental Disorders, University of Cape Town, Cape Town, South Africa; 9 MRC Clinical Trials Unit, University College London, London, UK; 10 SA MRC Unit On Child & Adolescent Health, University of Cape Town, Cape Town, South Africa

**Keywords:** HIV, Antiretroviral therapy, Brain structure, Neurodevelopment, Language, Magnetic resonance imaging

## Abstract

**Background:**

There is a growing population of children with in utero HIV exposure who are at risk of poor neurodevelopmental outcomes despite avoiding HIV infection. However, the underlying neurobiological pathways are not understood and neuroimaging studies are lacking. We aimed to investigate the cortical brain structure of children who are HIV-exposed and uninfected (HEU) compared to HIV-unexposed (HU) children and to examine the relationship with neurodevelopment.

**Methods:**

The Drakenstein Child Health birth cohort study enrolled pregnant women from a high HIV prevalence area in South Africa with longitudinal follow-up of mother–child pairs. High-resolution magnetic resonance imaging scans from 162 children (70 HEU; 92 HU) were acquired at 2–3 years of age. All HEU children were born to mothers taking antiretroviral therapy. Measures of brain structure (cortical thickness and surface area) in the prefrontal cortex regions were extracted from T1-weighted images and compared between groups using multivariate analysis of variance and linear regression. Child development, assessed using the Bayley Scales of Infant and Toddler Development-III, was correlated with cortical structure, and mediation analyses were performed.

**Results:**

Analyses demonstrated an association between HIV exposure and cortical thickness across the prefrontal cortex (*p* = 0.035). Children who were HEU had thicker cortices in prefrontal regions, with significantly greater cortical thickness in the medial orbitofrontal cortex (mOFC) bilaterally compared to HU children (3.21 mm versus 3.14 mm, *p* = 0.009, adjusted effect size 0.44 [95% CI 0.12 to 0.75]). Estimates held across multiple sensitivity analyses. There were no group differences in cortical surface area. Language scores, which were lower in HEU versus HU children (81.82 versus 86.25, *p* = 0.011, effect size − 0.44 [95% CI − 0.78 to − 0.09]), negatively correlated with prefrontal cortical thickness in both groups. Cortical thickness in the mOFC mediated the relationship between HIV exposure and poor language outcomes (Sobel test *p* = 0.032).

**Conclusions:**

In this cohort study, exposure to HIV during pregnancy was associated with altered cortical structure in early life. Our findings indicate that differences in cortical thickness development in the prefrontal region in children who are HEU may be a pathway leading to language impairment. Longitudinal studies are needed to determine the lasting impact.

**Supplementary Information:**

The online version contains supplementary material available at 10.1186/s12916-024-03282-6.

## Background

Antenatal HIV prevalence remains high in sub-Saharan Africa and in some countries over 20% of children are born to mothers living with HIV [1]. Substantial progress has been made in preventing vertical transmission of HIV with the scale-up of antiretroviral therapy (ART) in pregnancy. Alongside the decline in child HIV infections, the number of children with in utero HIV exposure who remain uninfected is increasing. Currently, there are an estimated 16 million children who are HIV-exposed and uninfected (HEU) worldwide [1]; therefore, any health problems associated with HIV exposure represent notable public health issues.

Children who are HEU have been reported to be at risk of impaired growth and neurodevelopment, particularly those living in low- and middle-income countries (LMICs) [2–7]. Early receptive and expressive language development have been shown to be particularly affected in cohorts from South Africa [8], Botswana [9, 10], and Zimbabwe [11]. A recent meta-analysis found HEU children had poorer expressive language development and gross motor function than HIV-unexposed (HU) children by 2 years of age [12]. Multiple factors may affect brain development during fetal and early life—sensitive periods of brain maturation—and contribute to impaired neurodevelopment in HEU children. These include exposure to HIV and an altered in utero environment, potential neurotoxic effects of ART exposure, and socioenvironmental factors associated with living in an HIV-affected household [3, 13, 14]. However, the neurobiological pathways underlying the association of in utero HIV exposure with poor neurodevelopment remain unclear.

Neuroimaging may be used to investigate neurodevelopmental pathways, in particular whether brain structural alterations underlie cognitive changes, and associations with disease processes [15, 16]. However, evidence is limited in children who are HEU and few studies have reported magnetic resonance imaging (MRI) in this population [17]. The scarce evidence suggests that HIV and/or ART exposure may affect brain development. Altered white matter microstructure in neonates and older children (7–10 years) has been reported in HEU compared to HU children, correlating with neurobehavioural function [18, 19]. Differences in neurometabolites between HEU and HU children have also been found [20, 21]. However, not all results are consistent [22]. In particular, structural imaging studies are lacking, although smaller total grey matter and subcortical brain volumes have been reported in neonates [23, 24]. Separately, individual antiretroviral drugs have been associated with adverse neurodevelopmental outcomes [10, 25], and animal models suggest a potential neurotoxic impact on the neocortex [26]. While qualitative brain imaging abnormalities were described in HEU children exposed to prior zidovudine monotherapy treatment [27], a recent study found that maternal triple ART through gestation may be protective for subcortical structures [24]. However, to our knowledge, no studies of children with in utero HIV and ART exposure have examined cortical surface area and thickness, core components of brain structure that are related to neurocognitive development [28, 29].

The Drakenstein Child Health Study (DCHS) is a South African population-based birth cohort that provides a unique opportunity to examine the brain structure of children who are HEU compared to demographically appropriate HIV-unexposed controls. Building upon our prior findings of increased language delay in HEU children [8], we aimed to compare the cortical neuroanatomy of children who are HEU and HU and to examine the structure–function relationship. We explore two main hypotheses: (1) in utero HIV exposure is associated with altered cortical structure and neurodevelopment at age 2–3 years and (ii) atypical patterns of structural brain development mediate the relationship between HIV exposure and neurodevelopmental function.

## Methods

### Study design and participants

This is a prospective neuroimaging study nested within the DCHS, a longitudinal birth cohort in a peri-urban area of the Western Cape, South Africa [30, 31]. The population is characterised by high levels of poverty and an antenatal HIV prevalence of 21% [32]. Mothers were enrolled between 2012 and 2015 from two public sector primary health care clinics at 20–28 weeks’ gestation while attending routine antenatal appointments. Eligibility criteria included age 18 years or older and intention to remain in the area attending one of the two clinics. Written informed consent was obtained at enrolment, and mothers are reconsented annually.

A sub-group of children aged between 2 and 3 years participated in the neuroimaging sub-study and were invited for an MRI scan between January 2016 and September 2018 following a small pilot [33]. Methods for child recruitment and neuroimaging are described in full elsewhere [33]. Briefly, children from the DCHS were eligible for neuroimaging if they resided in the study area, were aged 2–3 years, and did not have the following exclusion criteria: (i) medical comorbidity (genetic syndrome, neurological disorder, congenital abnormality); (ii) gestation < 36 weeks; (iii) Apgar score < 7 at 5 min; (iv) neonatal intensive care admission; (v) maternal use of illicit drugs during pregnancy; (vi) MRI contraindications; (vii) child HIV infection. All children with MRIs as neonates were invited for a scan at age 2–3 years, and additional children were selected for MRI to ensure adequate representation of risk factor exposure (including maternal HIV) along with a randomly selected comparison group [33]. Written informed consent was obtained from the parent/guardian at the neuroimaging visit.

### Study procedures

Mothers received routine HIV testing during pregnancy and the postnatal period following the Western Cape Prevention of Mother-to-Child Transmission (PMTCT) of HIV guidelines [34]. All HEU children had HIV-negative status confirmed through testing at 6 weeks using polymerase chain reaction (PCR) tests and at 9 and 18 months and post-cessation of breast-feeding using PCR, enzyme-linked immunosorbent assays, or rapid antibody testing as appropriate. Pregnant women living with HIV who were diagnosed before May 2013 received triple-drug ART or zidovudine monotherapy from 14 weeks’ gestation with nevirapine at delivery, based on WHO clinical stage and CD4 cell count, while those diagnosed after that point received triple-drug ART for life. HIV-exposed children received nevirapine prophylaxis alone or combined with zidovudine. HU children were defined as children born to HIV-uninfected mothers. Data on maternal ART use, infant prophylaxis, maternal CD4 cell count, and viral load data were collected from interviews, clinical notes, and the online National Health Laboratory Service system.

Sociodemographic data were collected at a baseline assessment during the third trimester of pregnancy using structured interviews and standardised questionnaires, and maternal smoking and alcohol use during pregnancy were also assessed [30, 31]. Maternal smoking was measured by self-report. Maternal alcohol use was assessed and quantified using the Alcohol, Smoking and Substance Involvement Screening Test (ASSIST) and retrospectively collected data on moderate-severe alcohol use in pregnancy forming a dichotomous measure [35]. Birth anthropometry measures were abstracted from hospital records. Weight and head circumference were measured at the scan and recorded using a standard protocol. Feeding data, including exclusive breastfeeding duration, were reported by mothers across multiple visits [32].

### Neurodevelopmental assessment

The Bayley Scales of Infant and Toddler development, third edition (BSID-III), was used to assess cognitive, language, and motor outcomes of children [35]. Two trained and experienced local assessors administered the BSID-III offering language prompts in the child’s preferred language, blinded to HIV exposure status. Assessors were monitored by a Paediatric Neurodevelopmental specialist to ensure reliability, accuracy, and standardised data collection. Age-adjusted composite scores were generated using normative values from a US reference population with a mean of 100 and a standard deviation of 15 [36]. Composite scores are standardised allowing comparison across ages and settings, and these have been validated in a South African setting [37]. Referral into appropriate clinical pathways for children with developmental delay was arranged.

### Neuroimaging

#### Image acquisition

Children were scanned during natural, non-sedated sleep to limit motion and scans were scheduled during typical sleep schedules using a child-friendly approach [33]. Once children were in a deep sleep, they were carefully positioned in the scanner with ear protection and stabilising cushions to reduce head movement during scans. High-resolution structural T1-weighted MR images were taken using a 3-Tesla Siemens Skyra 70-cm bore whole-body MRI scanner (Erlangen, Germany) at the Cape Universities Brain Imaging Centre, Groote Schuur Hospital, with a 32-channel head coil. A 3D MEMPRAGE (Multi-Echo Magnetization Prepared Rapid Acquisition Gradient Echo) sequence was used in sagittal orientation with the following image parameters: repetition time = 2530 ms; echo time = 1.69, 3.54, 5.39, 7.24 ms; inversion time = 1100 ms; flip angle = 7.0°; voxel size 1.0 × 1.0 × 1.0 mm^3^; field of view = 224 × 224 × 176 mm; 176 slices.

#### Image processing

Images were processed with FreeSurfer version 6.0 software [38], at the local supercomputing cluster at the Centre for High Performance Computing (CHPC, Cape Town). The cortex was parcellated into regions according to the Desikan-Killiany atlas [39] and measures of cortical structure (cortical thickness, mm, and surface area, mm^2^) were extracted for analysis (see Additional file 1: Text S1) [39–43].

#### Quality control

All structural sequences were reviewed by a radiologist blinded to HIV exposure status for incidental findings. Abnormal reports were referred for follow-up through appropriate local clinical pathways. Each scan was visually inspected for motion artefacts and for errors in segmentation processing following the standardised ENIGMA protocol [44].

#### Region selection

**Fig. 1:**
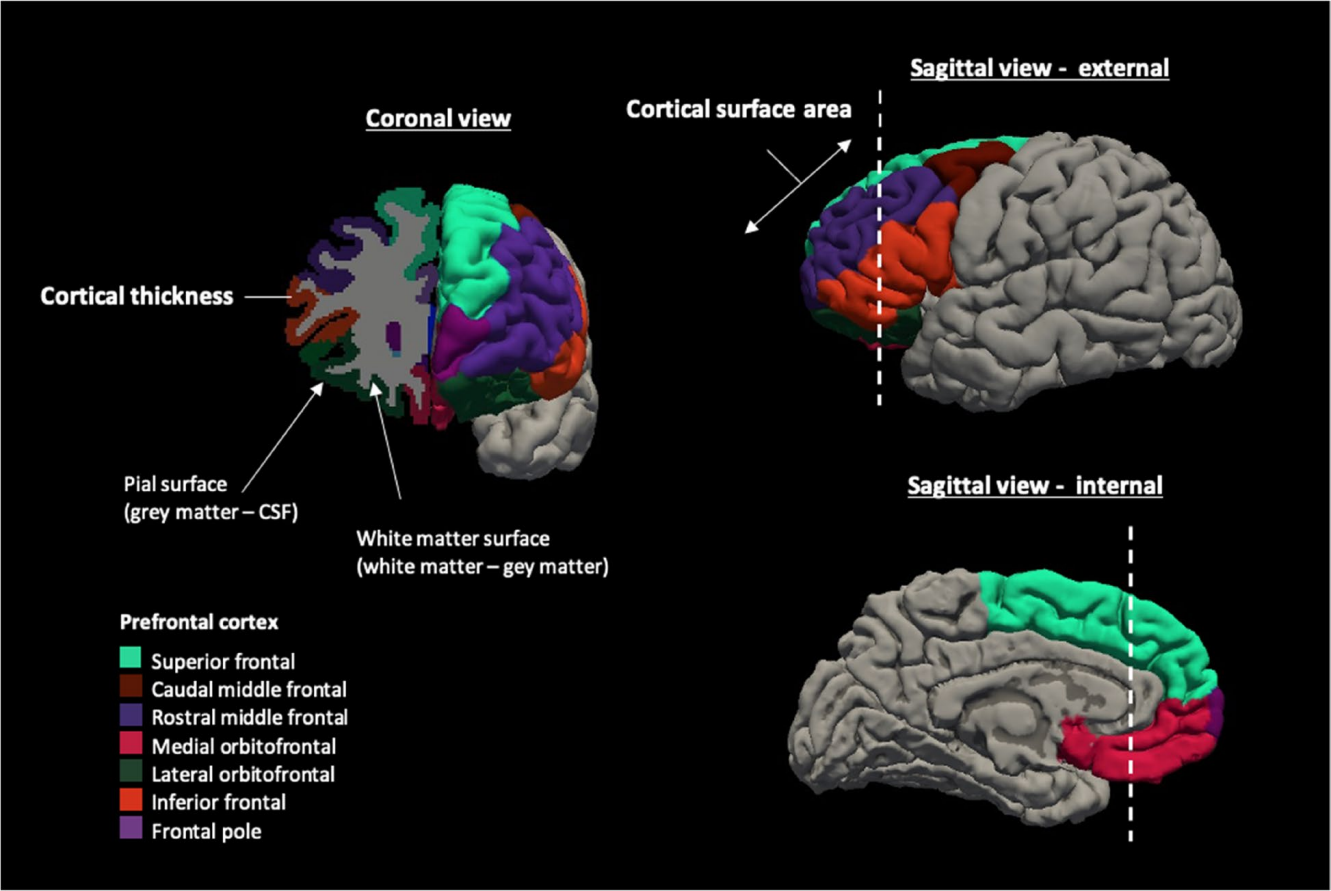
Schematic of the prefrontal cortex regions and the structural metrics of cortical brain structure. Cortical brain structure represents cortical thickness and cortical surface area. Cortical thickness describes the thickness of the layers of the cerebral cortex and is calculated as the distance from the white matter surface (white matter-grey matter boundary) to the pial surface (grey matter-CSF boundary). Cortical surface area and cortical thickness were calculated for each region. Regions visualised using a FreeSurfer template brain. Abbreviation: CSF, cerebrospinal fluid

Given reported neurodevelopmental impairment in children who are HEU [12], we hypothesised regions of the prefrontal cortex may be affected. Therefore, we conducted targeted analyses, expanding on prior exploratory findings showing an association between the frontal region and neurocognitive function in the early years [33]. All regions in the prefrontal cortex were selected a priori as they were determined to be biologically plausible areas due to their critical role in neurocognitive functioning and vulnerability to environmental exposures (further details may be found in Additional file 1: Text S1) [8, 16, 45–50]. For each participant, the mean values of the left and right hemispheres were used for analyses of each measure (cortical thickness, cortical surface area). The prefrontal regions are illustrated in Fig. 1, along with the components of cortical brain structure.

### Statistical analysis

Sociodemographic group differences were assessed using unpaired* t*-tests, chi-squared, or Fisher’s exact tests as appropriate between children who are HEU compared to HU. Comparisons were also made between the neuroimaging subgroup and the full DCHS cohort to assess generalisability.

To investigate group differences in cortical structure by HIV exposure, we first used multivariate analysis of variance to examine for a group-by-region effect in cortical surface area and cortical thickness separately. Pending a group-by-region effect, independent linear regression models were constructed to compare cortical structure in prespecified regions of interest (ROIs) between HEU and HU children. Partially adjusted multivariable linear models were created including child sex and child age as a priori confounders of interest [51]. Fully adjusted multivariable linear regression models were then created including additional confounders identified from the literature using a directed acyclic graph (DAG) including household income, maternal education, and age [8, 52]. We did not include characteristics that may be on the causal pathway. Where a significant association (*p* < 0.05) was identified, we examined each hemisphere separately using the same model. Mean differences and standardised effect sizes were reported using Cohen’s *d*. Normality of residuals and homogeneity of variance were checked in each model using quantile–quantile plots and scatterplots. Sensitivity analyses were performed to ensure our results were not impacted by alcohol use in pregnancy. Furthermore, we conducted restricted analyses of (i) the site where the majority of HEU children attended and (ii) limiting the HEU group to those exposed to the same maternal first-line ART regimen.

Neurodevelopmental outcomes were compared between groups using multivariable regression models described above, including cognitive, language, and motor composite scores as dependent variables. We then calculated Pearson’s correlation coefficients between cortical structure and neurodevelopmental outcomes for all ROIs in neurodevelopmental domains that showed a difference between HEU and HU groups (*p* < 0.05). We report correlation coefficients in the full sample and stratified by HIV exposure. We also calculated partial correlation coefficients adjusting for covariates as above. To explore evidence for effect modification by HIV exposure in regions with a significant correlation (*p* < 0.05), a linear regression model was fitted with neurodevelopment indices as dependent variables and the interaction between group and cortical variables.

Finally, we conducted a mediation analysis to test the hypothesis that associations between HIV exposure and neurodevelopmental outcomes are mediated by cortical brain structure. We applied the Baron and Kenny approach [53] that uses sequential regression analyses to test for mediation (see Additional file 1: Text S1). Models were adjusted for potential confounding variables identified a priori. We confirmed the results using structural equation modelling [54]. Statistical analyses were performed using STATA 14.2 (StataCorp Inc, College Station, TX, USA). *P* < 0.05 (two-tailed) was considered statistically significant.

## Results

### Demographics

**Fig. 2:**
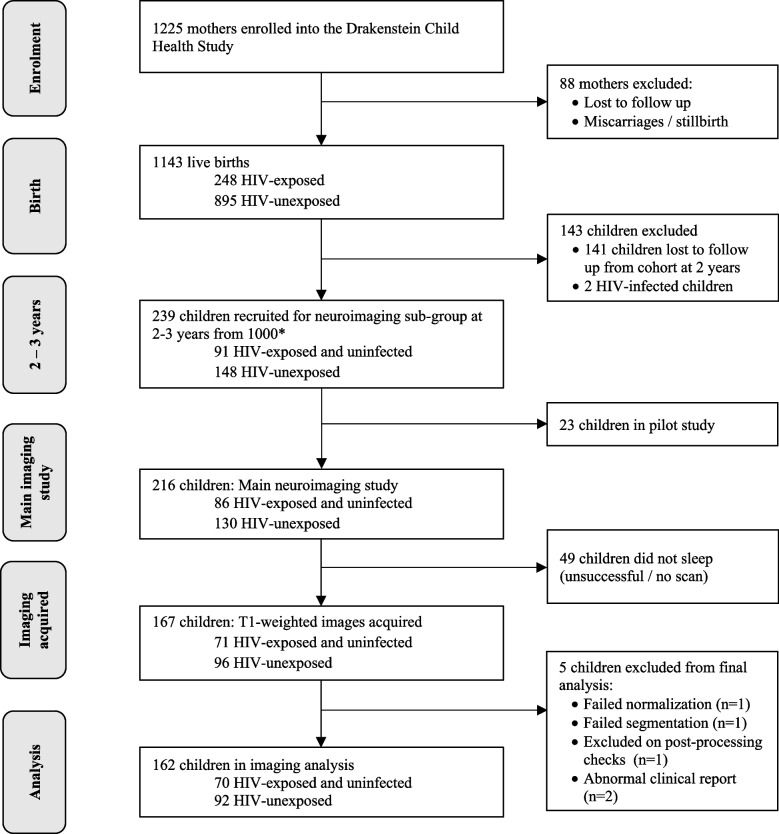
Drakenstein Child Health Study cohort flow chart of children with neuroimaging by HIV exposure. *Selection criteria for neuroimaging are fully described in methods. Inclusion criteria are as follows: (i) currently active in the cohort; (ii) residing in the study area; (iii) child aged 2–3 years. Exclusion criteria are as follows: (i) medical comorbidity (genetic syndrome, neurological disorder, or congenital abnormality); (ii) gestation < 36 weeks; (iii) low Apgar score (< 7 at 5 min); (iv) neonatal intensive care admission; (v) maternal use of illicit drugs during pregnancy; (vi) MRI contraindications; (vii) child HIV infection

A total of 1143 infants were born to 1137 women in the DCHS between May 2012 and September 2015. Two children were diagnosed with HIV infection and were not included in this analysis. Cohort retention was high, with 1000/1141 (87.6%) children in follow-up at 2 years. A sub-group of 216 children attended for MRI between January 2016 and September 2018 aged 2–3 years, following a small pilot (Fig. 2). High-resolution T1-weighted images were included for 162/216 (75%) children (70 HEU, 92 HU) excluding those children who did not sleep or scans that did not reach quality thresholds due to movement. Children in the neuroimaging sub-group were representative of the full cohort in socioeconomic variables (Additional file 1: Table S1).

Mothers and children in the two groups had similar demographic characteristics (Table 1). However, HEU children were born to mothers who were older, and a greater proportion were seen at Mbekweni clinic compared to TC Newman clinic. Children who were HEU had a trend for lower head circumference measurements at birth (*p* = 0.06), although this was not sustained at 2 years and other anthropometric measurements were comparable. Of the women living with HIV, 98.6% were taking triple-drug ART during pregnancy (97.1% non-nucleoside reverse transcriptase inhibitor-based ART; 1.4% protease inhibitor-containing ART) and 1.4% zidovudine monotherapy (Additional file 1: Table S2). Approximately half (39/70; 55.7%) of the mothers initiated ART during pregnancy. Median maternal CD4 was 476 cells/mm^3^, and most women with a viral load result had undetectable levels (77.2%) during pregnancy. The majority of children received postnatal prophylaxis with nevirapine alone (80.0%), versus nevirapine and zidovudine in the remainder.

**Table 1:** Sociodemographic and clinical characteristics by HIV exposure

Variable	Total *N* = 162	HEU children *N* = 70	HU children *N* = 92	*P*-value
*Sociodemographic characteristics*				
Child age at scan, months	34.1 (1.7)	33.8 (1.8)	34.3 (1.7)	0.096
Sex				
Female	68 (42.0%)	24 (34.3%)	44 (47.8%)	
Male	94 (58.0%)	46 (65.7%)	48 (52.2%)	0.084
Site				
TC Newman	48 (29.6%)	5 (7.1%)	43 (46.7%)	
Mbekweni	114 (70.4%)	65 (92.9%)	49 (53.3%)	< 0.001*
Monthly household income (ZAR)				
< R1000 (< ~ $75)	51 (31.5%)	24 (34.3%)	27 (29.4%)	
> R1000	111 (68.5%)	46 (65.7%)	65 (70.7%)	0.503
Maternal education				
Any secondary	108 (66.7%)	51 (72.9%)	57 (62.0%)	
Completed secondary	54 (33.3%)	19 (27.1%)	35 (38.0%)	0.145
Maternal employment status (employed)	44 (27.2%)	17 (24.3%)	27 (29.4%)	0.473
Maternal age at birth, years	28.0 (5.6)	29.6 (4.9)	26.8 (5.8)	0.002*
Maternal smoking during pregnancy	32 (19.8%)	9 (12.9%)	23 (25.0%)	0.054
Maternal alcohol use during pregnancy	24 (18.2%)	7 (13.5%)	17 (21.3%)	0.257
Duration of exclusive breastfeeding (months)	1.8 (1.8)	1.6 (2.1)	2.0 (1.5)	0.097
*Anthropometry*				
Birthweight, kg	3.1 (0.6)	3.0 (0.6)	3.1 (0.6)	0.291
Birth head circumference, cm	33.6 (2.0)	33.3 (1.9)	33.9 (2.0)	0.061
Weight at scan, kg	13.9 (1.9)	13.9 (2.0)	13.8 (1.9)	0.629
Head circumference at scan, cm	49.8 (1.8)	49.8 (1.8)	49.7 (1.8)	0.969
*Neuroanatomical variables*				
Total intracranial volume, mean (SD), cm^3^	1214 (118)	1208 (116)	1219 (119)	0.586
*Maternal and child HIV variables*				
Maternal CD4 in pregnancy, median (IQR) (cells/mm^3^)		476 (344—677)		-
< 350 cells/mm^3^		17 (27.9%)		-
350–500 cells/mm^3^		16 (26.2%)		-
≥ 500 cells/ mm^3^		28 (45.9%)		-
Maternal Viral load (VL) in pregnancy				
Lower than detectable (< 40 copies/mL)		44 (77.2%)		-
VL detectable (≥ 40–1000 copies/mL)		7 (12.3%)		-
Virally unsuppressed (> 1000 copies/mL)		6 (10.5%)		-
Timing of antiretroviral drug initiation				
Before conception		31 (44.3%)		-
During pregnancy		39 (55.7%)		-
Antiretroviral regimen during pregnancy				
Monotherapy with AZT [zidovudine]		1 (1.4%)		-
2 NRTIs + NNRTI [1st line]		68 (97.1%)		-
2 NRTIs + PI [2nd line]		1 (1.4%)		-
Infant prophylaxis				
NVP [nevirapine] alone		56 (80.0%)		-
NVP + AZT		14 (20.0%)		-

Data are* N* (%), mean (SD), or median (IQR). Continuous variables were compared with unpaired *t*-tests; categorical variables were compared with chi-squared tests. *p < 0.05. Percentages are cited among those with non-missing values. Missing data: alcohol (*n* = 30); birthweight (*n* = 1); head circumference at birth (*n* = 1) and at scan (*n* = 1); maternal CD4 (*n* = 9); maternal viral load (*n* = 13). The lowest maternal CD4 within 1 year prior to birth and 3 months post-birth was used to reflect maternal immunosuppression in pregnancy and maximise sample numbers. Maternal viral load was measured during pregnancy; where there was more than one result, the highest viral load was taken. Of the NRTI + 2NNRTIs, 64 mothers were taking efavirenz, emtricitabine/lamivudine, and tenofovir. *Abbreviations*: *HEU*, HIV-exposed and uninfected; *HU*, HIV-unexposed; *VL*, viral load; *NNRTI*, non-nucleoside reverse-transcriptase inhibitor; *NRTI*, nucleoside reverse transcriptase inhibitor; *PI*, protease inhibitor; *NVP*, nevirapine; *AZT*, zidovudine

### Neuroanatomy

Total intracranial volume was similar between child HEU (1208 cm^3^) and HU (1219 cm^3^) groups (*p* = 0.586). Multivariate group analysis showed a significant group-by-region effect for HIV exposure on cortical thickness measurements across the prefrontal cortex [*F* (7, 154) = 2.22, *p* = 0.035] but not for cortical surface area [*F* (7, 154) = 0.18, *p* = 0.989].

**Fig. 3:**
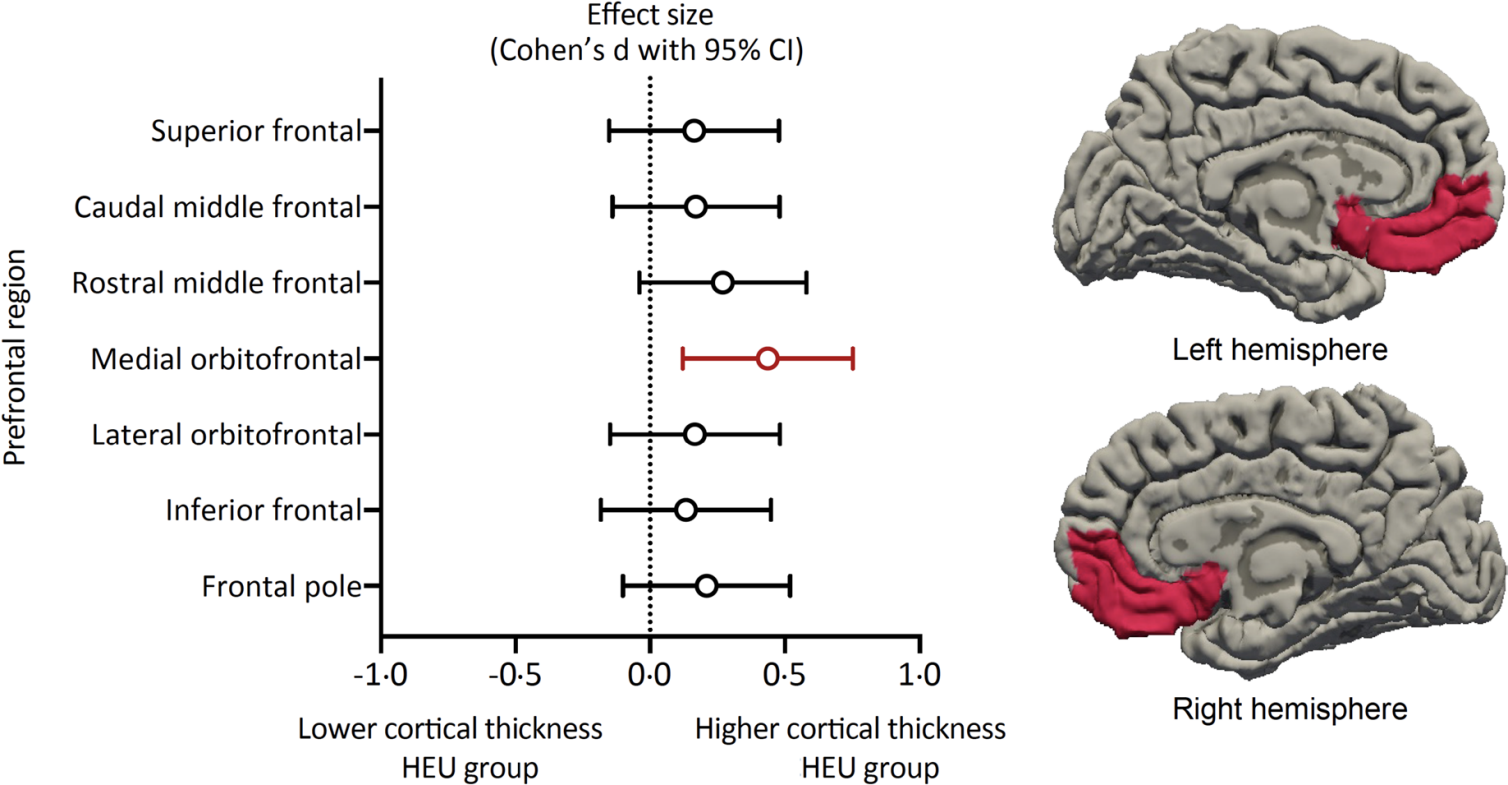
Associations of cortical thickness in anatomical regions of the prefrontal cortex with HIV exposure. Left panel: Figure displaying Cohen’s *d* effect sizes and 95% confidence intervals for cortical thickness differences between HEU and HU children across the prefrontal cortex regions. Effect sizes are shown after correction for child age and sex, household income, maternal age, and education. Positive values indicate higher cortical thickness in HEU children. Right panel: Regions with significantly increased cortical thickness in HEU children are highlighted in red on left and right medial views. Abbreviation: HEU, children who are HIV-exposed and uninfected

We therefore conducted further analyses on cortical thickness. Compared to HU children, HEU children had thicker cortices across all prefrontal cortex regions (Fig. 3). The model of the medial orbitofrontal cortex (mOFC) regions was statistically significant (Table 2). This held after adjusting for potential confounding variables with a moderate effect size (3.21 mm [HEU] versus 3.14 mm [HU], Cohen’s *d* 0.44 [95% confidence interval, CI 0.12 to 0.75], *p* = 0.009) (Table 2; Fig. 3). Post hoc tests demonstrated both hemispheres contributed to the overall mOFC effect (left mOFC effect size 0.36 [0.04 to 0.67]; right mOFC effect size 0.44 [0.12 to 0.75]).

**Table 2:** Adjusted mean differences in cortical thickness across the prefrontal cortex by HIV exposure groups

Prefrontal cortex regions	Cortical thickness (mm)		Minimally adjusted model^a^			Full adjusted model^b^		
	HEU Mean (SD) (*n* = 70)	HU Mean (SD) (*n* = 92)	Mean difference (95% CI)	*p*-value	Effect size	Mean difference (95% CI)	*p*-value	Effect size
Superior frontal	3.31 (0.16)	3.30 (0.16)	0.01 (− 0.04 to 0.06)	0.607	0.08 (− 0.23 to 0.39)	0.03 (− 0.03 to 0.08)	0.321	0.16 (− 0.15 to 0.48)
Caudal middle frontal	2.96 (0.16)	2.93 (0.15)	0.02 (− 0.03 to 0.07)	0.351	0.15 (− 0.16 to 0.46)	0.03 (− 0.02 to 0.08)	0.310	0.17 (− 0.14 to 0.48)
Rostral middle frontal	2.97 (0.12)	2.95 (0.13)	0.02 (− 0.02 to 0.06)	0.319	0.16 (− 0.15 to 0.47)	0.03 (− 0.01 to 0.07)	0.102	0.27 (− 0.04 to 0.58)
Medial orbitofrontal	3.21 (0.21)	3.14 (0.19)	0.08 (0.01 to 0.14)	0.016*	0.39 (0.07 to 0.70)	0.09 (0.02 to 0.15)	0.009*	0.44 (0.12 to 0.75)
Lateral orbitofrontal	3.27 (0.14)	3.26 (0.15)	0.01 (− 0.04 to 0.06)	0.648	0.07 (− 0.24 to 0.39)	0.02 (− 0.02 to 0.07)	0.318	0.17 (− 0.15 to 0.48)
Inferior frontal	3.15 (0.13)	3.15 (0.13)	0.00 (− 0.04 to 0.04)	0.923	0.02 (− 0.30 to 0.33)	0.02 (− 0.03 to 0.06)	0.412	0.13 (− 0.18 to 0.45)
Frontal pole	3.54 (0.27)	3.51 (0.30)	0.04 (− 0.05 to 0.13)	0.386	0.14 (− 0.17 to 0.45)	0.06 (− 0.03 to 0.15)	0.212	0.21 (− 0.10 to 0.52)

Multiple linear regression estimates for HIV exposure on cortical thickness. *p < 0.05. ^a^Adjusted for child age and sex. ^b^Adjusted for child age and sex, household income, maternal age, and education. Cortical thickness (mean of left and right hemispheres), mean differences (regression coefficients minimally and fully adjusted in multiple regression models), *p*-values, and effect sizes are presented. Effect sizes were calculated using Cohen’s *d* with associated 95% confidence intervals. Residuals were assessed for each model using quantile–quantile plots and scatterplots and were normally distributed. A positive mean difference estimate indicates that HIV exposure is associated with thicker cortex in that region. Post hoc, the regions with a significant difference were assessed bilaterally in fully adjusted models: medial orbitofrontal cortex (left hemisphere) effect size 0.36 (0.04 to 0.67) and (right hemisphere) effect size 0.44 (0.12 to 0.75). *Abbreviations: HEU*, HIV-exposed and uninfected; *HU*, HIV-unexposed

Our findings persisted across sensitivity analyses adjusting for self-reported alcohol use during pregnancy. We performed an analysis restricted to children from Mbekweni clinic to address between-site differences which revealed similar results, as did limiting the HEU group to those exposed to the same first-line regimen (efavirenz, emtricitabine/lamivudine, tenofovir) (Additional file 1: Table S3 and Table S4).

### Neurodevelopmental outcomes

Among children in the neuroimaging subgroup with neurodevelopmental data (*n* = 146), HEU children had lower composite language scores on BSID-III compared to HU children in minimal and fully adjusted analyses (81.82 versus 86.25, *p* = 0.011, adjusted Cohen’s *d* effect size − 0.44 [− 0.78 to − 0.09]), while cognitive and motor composite scores were similar (effect size − 0.21 [− 0.54 to 0.12] *p* = 0.228 and effect size − 0.09 [− 0.43 to 0.24] *p* = 0.602 respectively). Supplementary analyses with raw scores showed that differences in both receptive and expressive language were evident (Additional file 1: Table S5).

### Neuroanatomical regional associations with language function

**Fig. 4:**
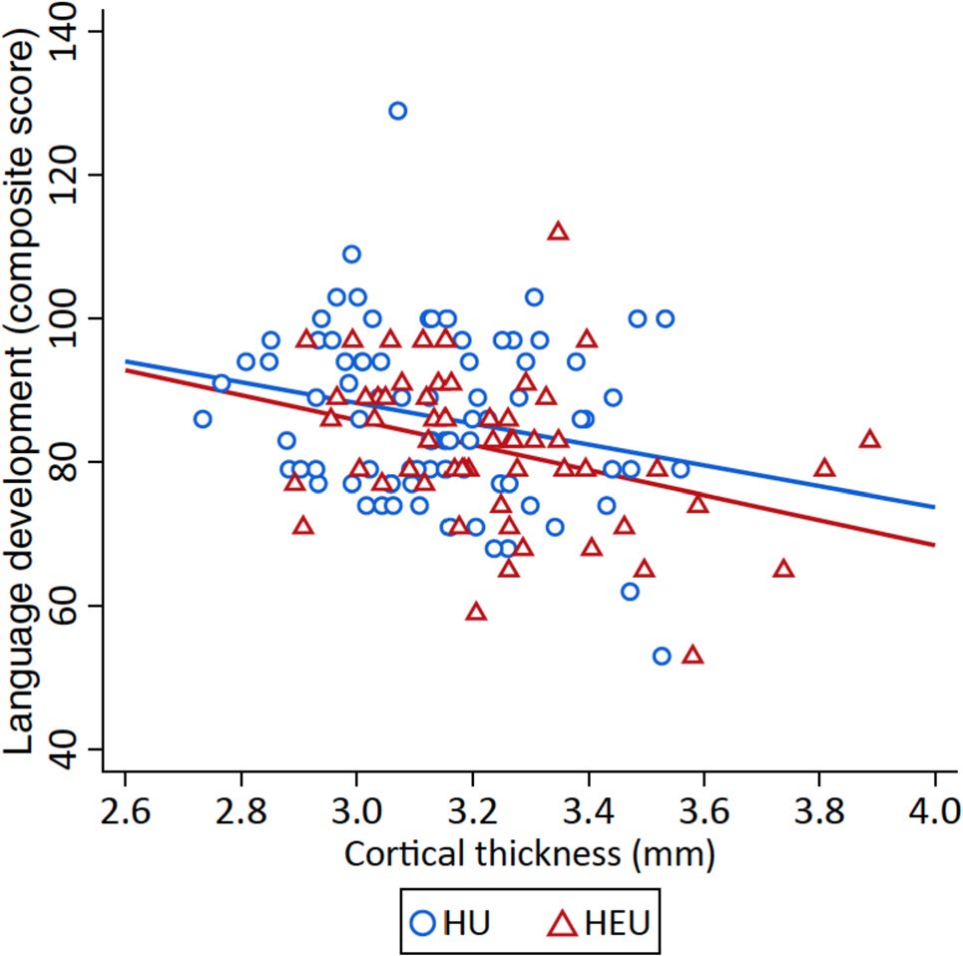
Linear regression of child language development by mOFC cortical thickness, stratified by HIV exposure. The relationship of child composite language score (BSID-III) with medial orbitofrontal region cortical thickness (mm) for HEU and HU children with line of best fit, *p* < 0.05. The direction of the correlation is negative, i.e. lower language scores are associated with a thicker cortex. Abbreviations: HEU, children who are HIV-exposed and uninfected; HU, children who are HIV-unexposed; mOFC, medial orbitofrontal cortex

Language development was negatively correlated with cortical thickness in multiple regions of the prefrontal cortex, most strongly with the mOFC (*r* =  − 0.31, *p* = 0.0002) (Additional file 1: Table S6). After adjusting for the full covariate set, only the correlation between language and mOFC thickness remained significant (*r* =  − 0.27, *p* = 0.002), and this was seen bilaterally (left mOFC: *r* =  − 0.22, *p* = 0.012; right mOFC *r* =  − 0.28, *p* = 0.001). When stratified by HIV exposure, correlations with mOFC thickness remained significant, and we found a stronger negative correlation in children who were HEU (*r* =  − 0.35, *p* = 0.008) compared to HU (*r* =  − 0.23, *p* = 0.038) (Fig. 4; Additional file 1: Table S6). However, on modelling the interaction effect, there was no effect modification by HIV exposure for the association between mOFC thickness and language outcomes (*p* = 0.759).

### Mediation analyses

**Fig. 5:**
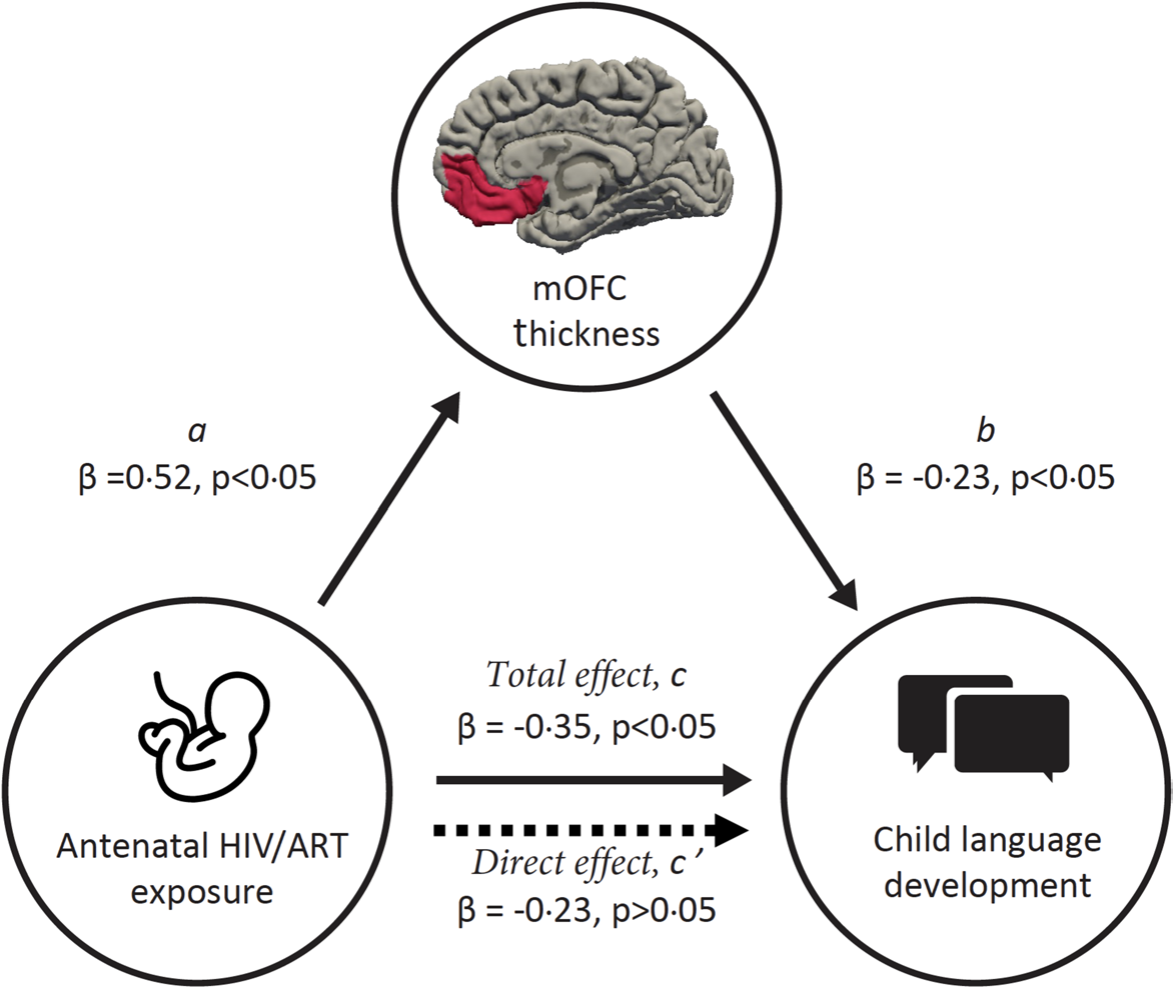
Illustration of mediation paths: mOFC thickness as a mediator between HIV exposure and child language. Estimates of the total (path *c*), direct (path *c’*), and indirect (path *ab*; mediated through the influence on structural brain development) effects of HIV exposure on child language. The proportion of the total effect of HIV exposure on child language mediated via mOFC thickness ≈ 35%, Sobel test* p* = 0.032. Results are displayed as standardised *β* regression coefficients adjusted for child age and sex, maternal age, education, and household income. Complete case analysis used *N* = 138 (*N* = 81 HEU, *N* = 57 HU). Abbreviations: mOFC, medial orbitofrontal cortex; HEU, HIV-exposed and uninfected; HU, HU-unexposed

Given the identified associations between in utero HIV exposure with cortical thickness and language outcomes, we conducted a mediation analysis. We found that increased cortical thickness in the mOFC mediated the observed association between HIV exposure and poor language outcomes through the Baron and Kenny approach [53] (significance testing of the indirect effect using the Sobel test: *p* = 0.032) (Fig. 5). We present estimates of the total and direct effects of HIV exposure on language development, alongside estimates of the indirect (mediated) effect that may be explained by the influence of HIV exposure on [adjusted] mOFC thickness. The proportion mediated through increased mOFC thickness was estimated to be 35%. This was further supported by structural equation modelling (Additional file 1: Table S7).

## Discussion

In this South African birth cohort, in utero HIV exposure was associated with altered cortical thickness in the prefrontal cortex at age 2–3 years. Language scores, which were lower in children who were HEU compared to HU, negatively correlated with cortical thickness in both groups. Overall, cortical thickness differences in the medial orbitofrontal region mediated approximately one third of the relationship between HIV exposure and language outcomes. Our results suggest that underlying changes in cortical brain structure may be one pathway leading to language impairment seen in children who are HEU, and specific prefrontal region functions may be disrupted at this age point.

Our findings that children with in utero HIV exposure had greater cortical thickness across all regions of the prefrontal cortex and significant cortical thickness differences in the mOFC bilaterally are novel. Few neuroimaging studies have been conducted in children who are HEU, and to our knowledge, cortical thickness and surface area have not been previously described. However, our results are consistent with reports using other imaging modalities showing that HIV and/or ART exposure may impact brain development. Previous studies have found lower subcortical volumes in early infancy in HEU compared to HU children [23, 24], differences in neurometabolites at 7 years [20], and altered white matter microstructure in neonates and older children associated with neurobehavioural function [17–19]. A limited number of studies of children with HIV infection have examined cortical modelling. Altered cortical thickness in 10–11-year-olds has been reported, including higher thickness in frontal and cingulate regions compared to controls [46].

Cortical thickness is a key component of brain structure, representing the number of neurons, synapses, glial, and dendritic processes connecting the layers of the neocortex [55]. Cortical thickness develops along an inverted U-shaped trajectory, increasing initially, peaking around 1–2 years, and then steadily decreasing through childhood as maturation occurs [16, 56]. The biological basis for the natural maturational thinning across the cortex is thought to reflect neuronal and synaptic pruning or myelination, leading to the formation of more organised and refined neural circuits [15, 56]. HEU children may therefore be demonstrating delayed cortical maturation or disrupted pruning or myelination at this early age. While the clinical significance of this remains to be determined, studies have found profiles of delayed cortical maturation may be associated with mood disorders [57], attention-deficit/hyperactivity disorder [58], and autism spectrum disorders [59], as well as being linked to other exposures including alcohol [60, 61] and infections such as cytomegalovirus infection (CMV) [62]. The process of cortical maturation is dynamic and there is substantial regional heterogeneity in the cortical thickness trajectory. Orbitofrontal regions are reported to peak the earliest (around 1 year) and decrease the fastest [15, 56]. We found that the medial orbitofrontal regions are the most affected in HEU children. This may reflect region-specific vulnerability or differences in the timing of development. Alternatively, the mOFC may be the earliest affected and other regions may become evident with time, highlighting the importance of longitudinal follow-up and serial imaging.

We found no evidence of differences in cortical surface area between HEU and HU children in our sample. Cortical thickness and surface area have heterogeneous temporal and regional patterns of development [15, 63], and while cortical thickness has been found to be influenced by environmental factors, surface area has stronger genetic links which may explain our findings [45]. Further, cortical thickness is largely determined in early life [45], whereas surface area is estimated to be 70% at 1 year and continues to increase [15]; therefore, changes may manifest later.

In this neuroimaging sub-group, HIV exposure was associated with lower language scores. Although there is heterogeneity across the broader literature with respect to neurodevelopment [12, 64], these findings are consistent with studies from multiple settings reporting worse language outcomes in HEU than in HU children [9, 11, 65], suggesting early language development may be at risk in children who are HEU. Further, we demonstrate that language development was negatively correlated with cortical thickness across multiple prefrontal regions. The most robust correlation was seen with the mOFC among both HEU and HU groups with a moderate effect size. It is well-established that the dynamic development of cognitive abilities parallels cortical maturation in early childhood [16, 28]. Our results are consistent with child development studies that have shown earlier cortical thinning in childhood is linked to the development of a more efficient network and better cognitive and language outcomes [28, 52], including verbal fluency [66].

Building on these findings, our mediation analysis demonstrated that approximately one third of the observed association between HIV exposure and language may be explained through an impact on mOFC cortical thickness. The results suggest that altered cortical maturation in frontal regions may partly underlie the reported language deficits. In early life, there is evidence to suggest a more widespread network of regions underly language function compared to later life [67, 68], with core input from higher-order centres, including the prefrontal cortex [69], until it becomes more automated [70]. The mOFC has previously been implicated in goal-directed behaviour, executive function, and reward processing [71, 72]. In the first few years, the rapid development and integration of sensory-motor and cognitive pathways in frontal regions which underly these functions may also contribute to language [73]. This has implications for later language functions such as sentence completion and story comprehension which have been associated with the mOFC [71].

Overall, we hypothesise that processes governing brain structural development in regions that support neurocognition may be disrupted in children who are HEU, impacting language outcomes. Several mechanisms may potentially impact cortical maturation processes of myelination and pruning related to perinatal HIV exposure including neuroinflammation, ART neurotoxicity, neurological infections such as CMV, and socioenvironmental factors [14, 16]. Given that 97% of mothers were on the same ART regimen (efavirenz, tenofovir, and emtricitabine) and most had suppressed viral loads, it is difficult to disentangle the contributions of HIV and ART. However, previous studies have highlighted an association between efavirenz and poorer receptive language [10] and microcephaly [25], and these need to be explored further. Separately, evidence from clinical and pre-clinical studies indicates that the immune system plays a critical role in brain development [74] including synaptic pruning [75], and maternal immune activation in pregnancy has been shown to alter prefrontal cortex morphology in particular [76]. Future studies should consider the impact of maternal immune function on brain development in this vulnerable group. Furthermore, given cortical structure mediated one third of the HIV-language relationship, ongoing exploration into the other contributing biological processes and mechanistic pathways linking HIV-specific and universal risk factors to HEU child neurodevelopmental outcomes is warranted.

There are several strengths of this study that add to the existing literature. This is the largest neuroimaging study to date to compare cortical brain structure between HEU and HU children during a critical period of brain growth. We explored cortical thickness and surface area in HEU children for the first time, the core components of cortical brain structure. We performed comprehensive neuroimaging and neurodevelopmental assessments blinded to HIV status minimising the risk of bias. Furthermore, this study was conducted amongst a well-characterised sample of HEU children representative of other high HIV-burden countries [77], with demographically appropriate controls, expanding generalisability from previous work.

There are limitations of the study that should be considered. Firstly, although our sample size was reasonably large for a neuroimaging study, we note that greater sample sizes are needed to withstand multiple comparisons across whole-brain analyses. We acknowledge the complexity and rapidly evolving nature of brain development, and further work is needed to explore other brain regions, including temporal, parietal, and subcortical structures, to understand wider network effects. Secondly, inherent challenges to MRI in young children mean we cannot rule out selection bias. However, mitigating these concerns, we established that sociodemographic characteristics were similar between children with and without imaging. Thirdly, as an observational study, this analysis does not establish causality. We measured neurodevelopment as standardised scores, with predictive validity across ages [78, 79], indicative of the child neurodevelopmental trajectory. However, longitudinal analyses are needed to confirm the relationship between structural and functional changes in children who are HEU at older ages. While the BSID-III has been validated for use in South Africa [37, 80], using a tool that is standardised in other populations is a limitation and there are reliability concerns regarding the use of US-normed data which may affect generalisability. We include a control group and share raw scores to add validity to our outcomes. Further standardisation is needed in SSA settings using contextually appropriate norms. Finally, although estimates held across multiple sensitivity analyses, other unmeasured confounding variables such as CMV infection may have resulted in residual bias, and there are likely multifactorial causal pathways. Further research would benefit from investigating potential mechanisms, in particular exploring the association with ART, including newer dolutegravir-based regimens which are now first-line treatment for HIV in pregnancy.

## Conclusions

In conclusion, we found altered patterns of cortical structure in children with in utero HIV exposure compared to demographically similar children without exposure at 2–3 years. Cortical thickness in the medial orbitofrontal cortex mediated the association between in utero HIV exposure and poor language outcomes. The findings suggest that HIV exposure may affect the maturation of prefrontal brain regions with implications for neurodevelopmental function. This has public health significance for the growing HEU population given brain development in early childhood is critical for long-term cognitive outcomes. However, it remains to be established whether these alterations persist, highlighting the need for ongoing neurodevelopmental surveillance and further studies to examine trajectories of brain maturation and underlying mechanisms.

### Supplementary Information

**Additional file 1: Text S1.** Detailed methods for image processing and analysis**. Table S1.** Comparison of study demographics of children with imaging versus those without imaging**. Table S2.** Antiretroviral drug regimens received by mothers with HIV during pregnancy**. Table S3.** Adjusted mean differences in cortical thickness according to HIV exposure restricted to one site**. Table S4.** Adjusted mean differences in cortical thickness according to HIV exposure restricted to HEU children born to mothers on the same first-line ART regimen**. Table S5.** Comparison of cognitive, language and motor development between HEU and HU children**. Table S6.** Correlations between cortical thickness and language development stratified by HIV exposure**. Table S7.** Structural equation model

## Data Availability

The de-identified data that support the findings of this study are available from the corresponding author upon reasonable request as per DCHS cohort guidelines.

## Abbreviations

ART: Antiretroviral therapy
ASSIST: Alcohol, Smoking and Substance Involvement Screening Test
BSID-III: Bayley Scales of Infant and Toddler Development, 3rd edition
CHPC: Centre for High Performance Computing
CI: Confidence interval
CMV: Cytomegalovirus
DAG: Directed acyclic graph
DCHS: Drakenstein Child Health Study
HEU: HIV-exposed uninfected
HIV: Human immunodeficiency virus
HU: HIV-unexposed
LMIC: Low- and middle-income countries
MEMPRAGE: Multi-Echo Magnetization Prepared Rapid Acquisition Gradient Echo
mOFC: Medial orbitofrontal cortex
MRI: Magnetic resonance imaging
ROI: Region-of-interest
PCR: Polymerase chain reaction
PMTCT: Prevention of mother-to-child transmission
WHO: World Health Organization
