# Supplementary material for: Association of in utero HIV exposure with child brain structure and language development: a South African birth cohort study
Source: BMC Med. 2024 Mar 22;22:129. doi: 10.1186/s12916-024-03282-6 (PMC10960435; doi:10.1186/s12916-024-03282-6)
Supplement: Supplementary file 1 — Additional file 1: Text S1. Detailed methods for image processing and analysis. Table S1. Comparison of study demographics of children with imaging versus those without imaging. Table S2. Antiretroviral drug regimens received by mothers with HIV during pregnancy. Table S3. Adjusted mean differences in cortical thickness according to HIV exposure restricted to one site. Table S4. Adjusted mean differences in cortical thickness according to HIV exposure restricted to HEU children born to mothers on the same first-line ART regimen. Table S5. Comparison of cognitive, language and motor development between HEU and HU children. Table S6. Correlations between cortical thickness and language development stratified by HIV exposure. Table S7. Structural equation model [file 12916_2024_3282_MOESM1_ESM.pdf]

**Association of *in utero* HIV exposure with child brain structure and language development: a South African birth cohort study**

# SUPPLEMENTARY INFORMATION

## Text S1: Detailed methods for image processing and analysis

### Image acquisition

A 3D MEMPRAGE (Multi-Echo Magnetization Prepared Rapid Acquisition Gradient Echo) sequence was used in sagittal orientation with repetition time (TR) = 2530ms, echo time (TE) = 1.69, 3.54, 5.39, 7.24 ms, inversion time (TI) = 1100ms, flip angle = 7.0°, voxel size 1.0x1.0x1.0 $mm^3$, field of view (FOV) = 224x224x176 mm, 176 slices, 1.0 mm thick. The overall duration was 5 minutes 21 seconds.

### Image processing

Images were processed with FreeSurfer version 6.0 software (http://surfer.nmr.mgh.harvard.edu/) at the local supercomputing cluster at the Centre for High Performance Computing (CHPC, Cape Town) (https://www.chpc.ac.za). The automated process includes motion correction, skull stripping, Talairach transformation, intensity normalization, volumetric segmentation and surface-based cortical reconstruction to produce anatomical measures . The cortex was parcellated into regions of interest (ROIs) according to the Desikan-Killiany atlas, and measures of cortical structure (cortical thickness, mm and surface area, $mm^2$) were extracted for analysis. Cortical thickness represents the shortest distance (mm) between the pial (grey-CSF boundary) and grey-white matter surfaces .

### Region-of-interest selection

All regions in the prefrontal cortex were selected *a priori* as they were determined to be biologically plausible areas due to their role in cognitive functioning. The prefrontal cortex is vulnerable to early environmental exposures and associations between the prefrontal cortex and neurocognitive development have been noted across ages. Studies of children with HIV suggest frontal regions are affected. Given documented neurocognitive delay in children who are HEU, we therefore hypothesised that regions in the prefrontal cortex would be affected. We used the automated Desikan-Killiany parcellation of the prefrontal cortex as described in prior papers and included all regions as follows: superior frontal, caudal middle frontal, rostral middle frontal, medial orbitofrontal, lateral orbitofrontal, inferior frontal gyrus (the pars triangularis, pars opercularis and pars orbitalis combined) and frontal pole. For each participant, the mean values of left and right hemispheres were used for subsequent analyses of each measure (cortical thickness, cortical surface area).

### Mediation Analyses

We performed a mediation analysis to evaluate whether, and to what extent, the effect of HIV exposure on language is mediated through cortical thickness – a hypothesis generated from findings in the prior analyses. Mediation analysis provides an estimation of the direct effect, indirect effect and total effect of HIV exposure (exposure) on language function (outcome), and whether the indirect effect is mediated by cortical thickness (mediator). We applied the Baron and Kenny (1986) approach that uses sequential regression analyses. The criteria for mediation are that there are associations between: (1) HIV exposure and language development (path *c*); (2) HIV exposure and cortical thickness (path *a*); (3) cortical thickness and language score (path *b*) controlling for the exposure; and that (4) the effect of HIV exposure on language function is lost (full mediation) or reduces (partial mediation) after controlling for cortical thickness (path *c'*). Models were adjusted for potential confounding variables identified *a priori*. The percentage that the mediator contributed was calculated as the ratio of the indirect effect coefficient to the total effect coefficient (proportion of total effect mediated). A reduction of the direct effect coefficient after adjusting for the mediator was taken as evidence of mediation. We confirmed the findings using structural equation modelling.

**Table S1: Comparison of study demographics of children with imaging versus those without imaging**

| | DCHS cohort[#] | | |
|---|---|---|---|
| | **Neuroimaging**<br>**162** | **No neuroimaging**<br>**979** | ***P*** |
| Male sex | 94 (58.0%) | 492 (50.3%) | 0.067 |
| Monthly household income (ZAR) | | | |
| < R1000 (<~$75) | 111 (68.5%) | 599 (61.2%) | 0.075 |
| >R1000 (>~$75) | 51 (31.5%) | 380 (38.8%) | |
| Maternal education | | | |
| Secondary | 108 (66.7%) | 586 (59.9%) | 0.100 |
| Completed secondary | 54 (33.3%) | 393 (40.1%) | |
| Maternal employment status (employed) | 44 (27.2%) | 263 (26.9%) | 0.937 |
| Birthweight, g | 3091 (574) | 3015 (607) | 0.134 |
| Birth head circumference, cm | 33.6 (2.0) | 33.5 (2.1) | 0.411 |
| Maternal smoking during pregnancy | 32 (19.8%) | 230 (23.7%) | 0.274 |
| Maternal alcohol use during pregnancy | 24 (18.2%) | 107 (12.4%) | 0.067 |
| Exclusive breastfeeding duration, months | 1.8 (1.8) | 2.1 (2.0) | 0.068 |

*Footnote*: Data are N(%), mean (SD). Continuous variables were compared with unpaired t-tests; categorical variables were compared with Chi-squared tests. #Full DCHS cohort excluding two children with HIV-infection. Percentages are cited among those with non-missing values. Missing data: birthweight (n=13); birth head circumference (n=23); maternal smoking (n=7); maternal alcohol use (n=146); breastfeeding (n=77).

## Table S2: Antiretroviral drug regimens received by mothers with HIV during pregnancy

| Antiretroviral drug (ARV) regimen | Child exposure N (%) |
|---|---|
| AZT monotherapy | 1 (1.4%) |
| 1st line three-drug ART (2 NRTIs + NNRTI) | |
| TDF + (3TC or FTC) + EFV (separately or fixed dose combination) | 64 (91.4%) |
| AZT + 3TC + EFV | 1 (1.4%) |
| TDF + FTC + NVP | 1 (1.4%) |
| AZT + 3TC + NVP | 2 (2.9%) |
| 2nd line ART (2 NRTIs + PI) | |
| AZT + 3TC + LPV/r | 1 (1.4%) |

*Footnote*: Abbreviations: ART = Antiretroviral therapy; AZT = zidovudine; EFV = efavirenz; 3TC = lamivudine; FTC = emtricitabine; TDF = tenofovir; NVP = nevirapine; LPV/r = lopinavir/ritonavir (Kaletra); NRTI = nucleoside reverse transcriptase inhibitor; NNRTI = non-nucleoside reverse transcriptase inhibitor; PI = protease inhibitor

**Table S3: Adjusted mean differences in cortical thickness according to HIV exposure restricted to one site**

| | Cortical thickness, mean SD) | | Minimally adjusted model[a] | | | Full adjusted model[b] | | |
|---|---|---|---|---|---|---|---|---|
| **Region** | HEU (n=65) | HU (n=49) | Mean difference (95% CI) | p-value | Effect size | Mean difference (95% CI) | p-value | Effect size |
| Superior frontal | 3.31 (0.16) | 3.28 (0.17) | 0.02 (-0.04 to 0.09) | 0.471 | 0.14 (-0.23 to 0.51) | 0.03 (-0.03 to 0.10) | 0.343 | 0.19 (-0.18 to 0.56) |
| Caudal middle frontal | 2.96 (0.16) | 2.95 (0.15) | 0.00 (-0.06 to 0.06) | 0.907 | 0.02 (-0.35 to 0.39) | 0.01 (-0.06 to 0.07) | 0.845 | 0.04 (-0.33 to 0.41) |
| Rostral middle frontal | 2.97 (0.12) | 2.95 (0.13) | 0.00 (-0.04 to 0.05) | 0.930 | 0.02 (-0.35 to 0.39) | 0.01 (-0.04 to 0.06) | 0.665 | 0.08 (-0.29 to 0.45) |
| Medial orbitofrontal | 3.22 (0.22) | 3.12 (0.18) | 0.10 (0.02 to 0.17) | 0.011* | 0.49 (0.11 to 0.86) | 0.10 (0.02 to 0.18) | 0.015* | 0.49 (0.11 to 0.86) |
| Lateral orbitofrontal | 3.26 (0.14) | 3.24 (0.13) | 0.02 (-0.03 to 0.08) | 0.415 | 0.16 (-0.21 to 0.53) | 0.03 (-0.03 to 0.08) | 0.286 | 0.21 (-0.16 to 0.58) |
| Inferior frontal | 3.14 (0.13) | 3.14 (0.14) | -0.00 (-0.06 to 0.05) | 0.893 | -0.03 (-0.40 to 0.34) | 0.01 (-0.05 to 0.06) | 0.827 | 0.04 (-0.33 to 0.41) |
| Frontal pole | 3.52 (0.26) | 3.49 (0.27) | 0.02 (-0.08 to 0.12) | 0.746 | 0.06 (-0.31 to 0.43) | 0.03 (-0.08 to 0.13) | 0.589 | 0.11 (-0.26 to 0.48) |

*Footnote*: Multiple linear regression estimates for HIV exposure on cortical thickness (mm) restricted to the clinic site where the majority of HEU children attend. *p<0·05 [a]Adjusted for child age and sex; [b]Adjusted for child age and sex, household income, maternal age and education. Cortical thickness (mean of left and right hemispheres), mean differences (regression coefficients minimally and fully adjusted in multiple regression models), p-values and effect sizes are presented. Effect sizes were calculated using Cohen's d with associated 95% confidence intervals. Residuals were assessed for each model using quantile-quantile plots and were normally distributed. A positive regression estimate indicates HIV exposure is associated with thicker cortices in that region. Abbreviations: HEU = children who are HIV-exposed and uninfected; HU = children who are HIV-unexposed.

## Table S4: Adjusted mean differences in cortical thickness according to HIV exposure restricting to HEU children born to mothers on the same first-line ART regimen

| | Minimally adjusted model[a] | | | Full adjusted model[b] | | |
|---|---|---|---|---|---|---|
| **Region** | Mean difference (95% CI) | p-value | Effect size | Mean difference (95% CI) | p-value | Effect size |
| Superior frontal | 0.01 (-0.04 to 0.07) | 0.592 | 0.09 (-0.23 to 0.41) | 0.03 (-0.03 to 0.08) | 0.311 | 0.17 (-0.15 to 0.49) |
| Caudal middle frontal | 0.02 (-0.03 to 0.07) | 0.457 | 0.12 (-0.19 to 0.44) | 0.02 (-0.03 to 0.08) | 0.397 | 0.15 (-0·17 to 0.47) |
| Rostral middle frontal | 0.02 (-0.02 to 0.06) | 0.281 | 0.18 (-0.14 to 0.50) | 0.04 (-0.00 to 0.08) | 0.081 | 0.30 (-0·02 to 0.62) |
| Medial orbitofrontal | 0.08 (0.01 to 0.14) | 0.020* | 0.39 (0.06 to 0.71) | 0.09 (0.02 to 0.15) | 0.011* | 0.44 (0·12 to 0.76) |
| Lateral orbitofrontal | 0.02 (-0.03 to 0.07) | 0.426 | 0.13 (-0.19 to 0.45) | 0.03 (-0.01 to 0.08) | 0.163 | 0.24 (-0.08 to 0.56) |
| Inferior frontal | 0.00 (-0.04 to 0.05) | 0.854 | 0.03 (-0.29 to 0.35) | 0.02 (-0.02 to 0.07) | 0.356 | 0.16 (-0.16 to 0.48) |
| Frontal pole | 0.04 (-0.05 to 0.14) | 0.368 | 0.15 (-0.17 to 0.47) | 0.06 (-0.03 to 0.16) | 0.196 | 0.23 (-0.09 to 0.55) |

*Footnote*: Multiple linear regression estimates for HIV exposure on cortical thickness (mm) restricting HEU to children born to mothers on the same WHO recommended first-line ART regimen (efavirenz + emtricitabine/lamivudine + tenofovir) (n=64); total n=156. *p<0.05
[a]Adjusted for child age and sex; [b]Adjusted for child age and sex, household income, maternal age and education. Cortical thickness (mean of left and right hemispheres), mean differences (regression coefficients minimally and fully adjusted in multiple regression models), p-values and effect sizes are presented. Effect sizes were calculated using Cohen's d with associated 95% confidence intervals. A positive regression estimate indicates HIV exposure is associated with thicker cortices in that region. Abbreviations: HEU = children who are HIV-exposed and uninfected.

## Table S5: Comparison of cognitive, language, and motor development between HEU and HU children

| BSID-III domain (composite scores) | Composite Score Mean (SD) | | Minimally adjusted model[a] | | | Fully adjusted model[b] | | |
|---|---|---|---|---|---|---|---|---|
| | HEU | HU | Mean difference | p-value | Effect size | Mean difference | p-value | Effect size |
| Cognition | 85.00 (9.30) | 87.44 (9.29) | -2.36 (-5.48 to 0.77) | 0.138 | -0.25 (-0.58 to 0.08) | -1.98 (-5.22 to 1.26) | 0.228 | -0.21 (-0.54 to 0.12) |
| Language | 81.82 (10·68) | 86.25 (11.84) | -4.52 (-8.43 to -0.62) | 0.024* | -0.38 (-0.73 to -0.04) | -5.16 (-9.13 to -1.18) | 0.011* | -0.44 (-0.78 to -0.09) |
| Motor | 93.22 (11.38) | 94.47 (10.55) | -1.44 (-5.17 to 2.29) | 0.446 | -0.13 (-0.47 to 0.20) | -1.03 (-4.93 to 2.87) | 0.602 | -0.09 (-0.43 to 0.24) |

| BSID-III domain (raw Scores) | Raw Score Mean (SD) | | Minimally adjusted model[a] | | | Fully adjusted model[b] | | |
|---|---|---|---|---|---|---|---|---|
| | HEU | HU | Mean difference | p-value | Effect size | Mean difference | p-value | Effect size |
| Cognition | 55.38 (4.97) | 56.64 (4.61) | -1.27 (-2.87 to 0.32) | 0.116 | -0.26 (-0.60 to 0.07) | -1.14 (-2.79 to 0.512) | 0.175 | -0.24 (-0.57 to 0.09) |
| Receptive language | 19.93 (3.07) | 21.28 (3.76) | -1.36 (-2.51 to -0.21) | 0.021* | -0.38 (-0.71 to -0.04) | -1.57 (-2.75 to -0.39) | 0.010* | -0.43 (-0.77 to -0.10) |
| Expressive language | 23.23 (5.69) | 25.00 (5.26) | -1.90 (-3.74 to -0.07) | 0.042* | -0.34 (-0.68 to -0.00) | -2.22 (-4.05 to -0.38) | 0.018* | -0.40 (-0.74 to -0.05) |
| Fine motor | 37.57 (3.22) | 37.91 (3.28) | -0.37 (-1.44 to 0.71) | 0.500 | -0.11 (-0.44 to 0.22) | -0.23 (-1.34 to 0.88) | 0.688 | -0.07 (-0.40 to 0.26) |
| Gross motor | 53.72 (2.89) | 53.67 (3.09) | -0.05 (-1.07 to 0.76) | 0.926 | -0.02 (-0.35 to 0.32) | -0.02 (-1.10 to 1.06) | 0.968 | -0.01 (-0.34 to 0.33) |

*Footnote*: Composite neurodevelopmental domain scores, mean differences (regression coefficients minimally and fully adjusted in multiple regression models), p-values and effect sizes for associations between neurodevelopment and HIV exposure. Supplementary table with raw scores below. [a]Adjusted for child age and sex; [b]Adjusted for child age and sex, household income, maternal age and education. *p<0·05. Effect sizes were calculated using Cohen's d with associated 95% confidence intervals. Residuals were assessed for each model using quantile-quantile plots and were normally distributed. A negative regression estimate indicates HIV exposure is associated with lower scores in that domain. Cognitive n=146 (HEU 60, HU 86); Language n=138 (HEU 57, HU 81); Motor n=141 (HEU 58, HU 83). Abbreviations: HEU = children who are HIV-exposed and uninfected; HU = children who are HIV-unexposed.

## Table S6: Correlations between cortical thickness and language development stratified by HIV exposure

| | Pearson's correlations | | | | | |
|---|---|---|---|---|---|---|
| | Total | | HEU | | HU | |
| **Region** | ***r*** | ***p*** | ***r*** | ***p*** | ***r*** | ***p*** |
| Superior frontal | -0.17 | 0.041* | -0.25 | 0.057 | -0.10 | 0.388 |
| Caudal middle frontal | -0.17 | 0.046* | -0.16 | 0.242 | -0.14 | 0.223 |
| Rostral middle frontal | -0.19 | 0.028* | -0.18 | 0.175 | -0.14 | 0.211 |
| Medial orbitofrontal | -0.31 | 0.0002* | -0.35 | 0.008* | -0.23 | 0.038* |
| Lateral orbitofrontal | -0.14 | 0.112 | -0.14 | 0.312 | -0.11 | 0.317 |
| Inferior frontal | -0.18 | 0.031* | -0.14 | 0.305 | -0.19 | 0.084 |
| Frontal pole | -0.08 | 0.366 | 0.03 | 0.821 | -0.11 | 0.344 |

*Footnote*: Pearson's correlations between language development and HIV exposure. *p<0·05. Total n=138 (HEU 57; HU 81). Abbreviations: HEU = children who are HIV-exposed and uninfected; HU = children who are HIV-unexposed; mOFC = medial orbitofrontal cortex.

**Table S7: Structural equation model**

| Variable (n=138) | |
|---|---|
| Direct effects | **β, 95% CI, *p*-value** |
| HIV exposure → mOFC thickness *(path a)* | 0.52 (0.18 to 0.86), p=0.003 |
| mOFC thickness → Language *(path b)* | -0.23 (-0.39 to -0.07), p=0.004 |
| HIV exposure → Language *(path c)* | -0.23 (-0.56 to 0.10), p=0.177 |
| Indirect effects | |
| HIV exposure → Language *(ab)* | -0.12 (-0.23 to -0.01), p=0.038 |
| Total effects | |
| HIV exposure → mOFC thickness | 0.52 (0.18 to 0.86), p=0.003 |
| mOFC thickness → Language | -0.23 (-0.39 to -0.07), p=0.004 |
| HIV exposure → Language *(ab + c)* | -0.35 (-0.68 to -0.02), p=0.039 |

*Covariates included in models: child age and sex, household income, maternal age and education

Proportion of total effect mediated: indirect/total effect: 0.35
Ratio of indirect to direct effect: indirect/direct: 0.53
Ratio of total to direct effect: total/direct: 1.53

**Summary:** Estimates of the direct and indirect (mediated through brain structure) effect of HIV exposure on language development, measured by the BSID-III are shown. All scores were standardized. The total effect for HIV exposure on language development is -0.35. The direct effect for HIV exposure on language development is smaller than the total effect (-0.23). The indirect effect of HIV that passes through the mediator, mOFC thickness, is -0.12, which is statistically significant. The proportion of the total effect that is mediated is approximately one third (35%), and the ratio of the indirect effect:direct effect is approximately half the size of the direct effect (0.53). Similar results are obtained in unadjusted analyses, holding on bootstrapping with 1000 repetitions. Significance testing of indirect effect (adjusted, standardized) using the Sobel method showed mediation is complete (p=0.032). Similar results were obtained using the Monte Carlo test. Abbreviation: mOFC: medial orbitofrontal cortex
